# A Pilot Study of Low-Dose Craniospinal Irradiation in Patients With Newly Diagnosed Average-Risk Medulloblastoma

**DOI:** 10.3389/fonc.2021.744739

**Published:** 2021-09-02

**Authors:** Jane E. Minturn, Aaron Y. Mochizuki, Sonia Partap, Jean B. Belasco, Beverly J. Lange, Yimei Li, Peter C. Phillips, Iris C. Gibbs, Paul G. Fisher, Michael J. Fisher, Anna J. Janss

**Affiliations:** ^1^Department of Pediatrics, Division of Oncology, The Children’s Hospital of Philadelphia, Philadelphia, PA, United States; ^2^Department of Pediatrics, Division of Oncology, Cincinnati Children’s Hospital Medical Center, Cincinnati, OH, United States; ^3^Department of Neurology and Neurological Sciences, Division of Child Neurology, Lucile Packard Children’s Hospital at Stanford University, Palo Alto, CA, United States; ^4^Department of Radiation Oncology, Stanford University Cancer Center, Palo Alto, CA, United States; ^5^Department of Pediatrics, Division of Hematology/Oncology, Children’s Healthcare of Atlanta, Atlanta, GA, United States

**Keywords:** medulloblastoma, craniospinal irradiation (CSI), neurotoxicity, late effects after cancer therapy, survivorship

## Abstract

**Purpose:**

Medulloblastoma is one of the most common malignant brain tumors in children. To date, the treatment of average-risk (non-metastatic, completely resected) medulloblastoma includes craniospinal radiation therapy and adjuvant chemotherapy. Modern treatment modalities and now risk stratification of subgroups have extended the survival of these patients, exposing the long-term morbidities associated with radiation therapy. Prior to advances in molecular subgrouping, we sought to reduce the late effects of radiation in patients with average-risk medulloblastoma.

**Methods:**

We performed a single-arm, multi-institution study, reducing the dose of craniospinal irradiation by 25% to 18 Gray (Gy) with the goal of maintaining the therapeutic efficacy as described in CCG 9892 with maintenance chemotherapy.

**Results:**

Twenty-eight (28) patients aged 3-30 years were enrolled across three institutions between April 2001 and December 2010. Median age at enrollment was 9 years with a median follow-up time of 11.7 years. The 3-year relapse-free (RFS) and overall survival (OS) were 79% (95% confidence interval [CI] 58% to 90%) and 93% (95% CI 74% to 98%), respectively. The 5-year RFS and OS were 71% (95% CI 50% to 85%) and 86% (95% CI 66% to 94%), respectively. Toxicities were similar to those seen in other studies; there were no grade 5 toxicities.

**Conclusions:**

Given the known neurocognitive adverse effects associated with cranial radiation therapy, studies to evaluate the feasibility of dose reduction are needed. In this study, we demonstrate that select patients with average-risk medulloblastoma may benefit from a reduced craniospinal radiation dose of 18 Gy without impacting relapse-free or overall survival.

**Clinical Trial Registration:**

ClinicalTrials.gov identifier: NCT00031590

## Introduction

Medulloblastoma is the most common malignant brain tumor in children, with an annual incidence of 0.4 per 100,000 population aged 0-19 years (1). Optimal treatment of average-risk (i.e., non-metastatic, gross totally resected) medulloblastoma includes surgical resection, craniospinal irradiation (CSI) and chemotherapy. Over the past several decades, studies have aimed to mitigate the adverse neurocognitive effects of radiation by reducing the craniospinal radiation dose. In a clinical trial from the Children’s Cancer Group (CCG) and Pediatric Oncology Group (POG) for low-stage (gross totally resected tumor with no metastasis and no brainstem invasion) medulloblastoma that randomized patients to 23.4 Gy or 36 Gy CSI without adjuvant chemotherapy, early relapses and increased exoprimary failure rate were seen in the lower radiation dose arm (2). Studies that incorporated adjuvant chemotherapy in average-risk medulloblastoma demonstrated 5-year event free survival (EFS) over 80% in the late 1990s (3, 4). The increased survival of children with medulloblastoma into adulthood revealed the potentially severe late effects and resultant decreased quality of life that may occur as a consequence of radiation therapy (5–8). Thus, attempts have been made to further reduce CSI doses in lower risk patients (2, 9, 10). In the Children’s Oncology Group trial ACNS0331 children ages 3 to 7 years diagnosed with medulloblastoma were randomized to 18 Gray (Gy) or 23.4 Gy CSI with variable posterior fossa radiation boosts, followed by nine cycles of alkylator-based chemotherapy (11).

In this single-arm, multi-institution pilot study, we aimed to reduce the late neurotoxic (otologic, neuroendocrine, neurocognitive, neurovascular) effects of treatment in patients with average-risk medulloblastoma across a broader age range from 3 to 30 years at diagnosis. To do so, the craniospinal irradiation dose was decreased to 18 Gy while maintaining the standard chemotherapy as described in CCG 9892 (12) and adding cyclophosphamide and etoposide as in the contemporaneous trials CCG 9961 (13) and POG 9631 (14), respectively.

## Methods

### Patients

Eligible patients were age 3 to 30 years at the time of diagnosis with average-risk, histologically proven medulloblastoma as determined by each participating institution. Average-risk was defined as less than 1.5 cm^3^ residual tumor following resection by neuroimaging, no evidence of metastases by brain and spine MRI, and no tumors cells present on cerebrospinal fluid (CSF) cytology. CSF was obtained by lumbar puncture between 10 days and 4 weeks after surgery. Postoperative MRI of the primary site with and without contrast was required within 72 hours of surgery, preferably within 48 hours; volumetrics were performed by neuroradiologists at their respective institutions.

### Study Design

This single-arm study was developed by the authors and conducted at three institutions in the United States. The primary objective was to reduce the late toxicity of CSI without compromising the therapeutic efficacy compared with standard therapy [86% ± 4% standard error of the mean (SEM) three-year relapse-free survival per CCG 9892 (12)]. The secondary objectives were to assess relapse-free survival and overall survival 5 years from time of study enrollment.

Surgical resection was carried out according to institutional standards with staging as stated above. Following institutional diagnosis of medulloblastoma, histologic slides were sent to the Children’s Hospital of Philadelphia for correlative studies.

Patients began CSI within four weeks of surgical resection; concurrent weekly intravenous (IV) vincristine 1.5mg/m^2^ (maximum single dose 2 mg) was administered for 6 doses. Radiation therapy consisted of 18 Gy photons to the whole brain and spine in 10 fractions with a boost to the tumor bed in 21 fractions for a total dose of 55.8 Gy to the tumor bed. Four weeks after completion of radiotherapy, maintenance chemotherapy was initiated, consisting of 9 cycles of regimens A and B in an AABAABAAB pattern. Regimen A included oral lomustine 75 mg/m^2^ and IV cisplatin 70 mg/m^2^ on day 0, and IV vincristine 1.5 mg/m^2^ (maximum single dose 2 mg) on days 0, 7, and 14. For regimen B, patients received IV cyclophosphamide 1000 mg/m^2^ on days 0 and 1 and IV etoposide 150 mg/m^2^ on days 0 and 1, followed by oral etoposide 50 mg/m^2^ on days 14-34.

All patients were monitored for adverse events according to the National Cancer Institute Common Terminology Criteria for Adverse Events, version 3.0. MRI of the brain and spine with and without gadolinium was obtained prior to surgery, post-operatively, in 3-month intervals during maintenance chemotherapy and in the first year after the end of treatment. Participants then underwent imaging every 6 months for years 2 and 3, and yearly thereafter or if clinically indicated for suspected relapse. Relapse was defined as a greater than 25% increase in the product of the longest two perpendicular measurements of residual tumor on MRI, evidence of new tumor by MRI, or *de novo* tumor cells in the CSF. Patients were removed from study for relapse, unacceptable toxicity or other extraordinary medical circumstances, inability or refusal to comply with study treatment or follow up with required observations, or death.

Audiograms were obtained prior to the start of each chemotherapy cycle, every 6 months from one to three years after the completion of chemotherapy and yearly thereafter. Laboratory studies to evaluate liver, kidney and endocrinologic function as well as MRI were obtained serially according to study protocol. Neurocognitive testing utilizing age-appropriate Wechsler Scales was obtained within 6 months of diagnosis and at one, two and three years after the completion of chemotherapy.

The study was approved by institutional review boards at the participating institutions and conducted in accordance with the Declaration of Helsinki. All patients or their legal guardians provided written informed consent and age-appropriate assent was obtained. ClinicalTrials.gov identifier: NCT00031590.

### Statistical Analysis

The target accrual of the study was 50 patients over 3 years, assuming that 15% represents an acceptable difference in relapse-free survival between the study and the standard survival rate of 86 ± 4% at 3 years in CCG 9892 (12). With a sample size of 50, 11 events would represent relapse-free survival of 78% with a 90% confidence interval (CI) of 70.8% - 85.8%. Stopping rules were designed to close accrual if local relapses, exoprimary relapses or toxicity of the protocol exceeded those reported in CCG 9892 (12). Relapse-free survival (RFS) was defined as the time to first disease progression, disease recurrence, death from any cause, or occurrence of a subsequent malignant neoplasm. Overall survival (OS) was defined as time to death from any cause. Time to first relapse was defined as the time from diagnosis to the time of tumor relapse as previously defined. RFS and OS and were calculated using the methods of Kaplan and Meier.

## Results

### Patient Characteristics

**Figure 1:**
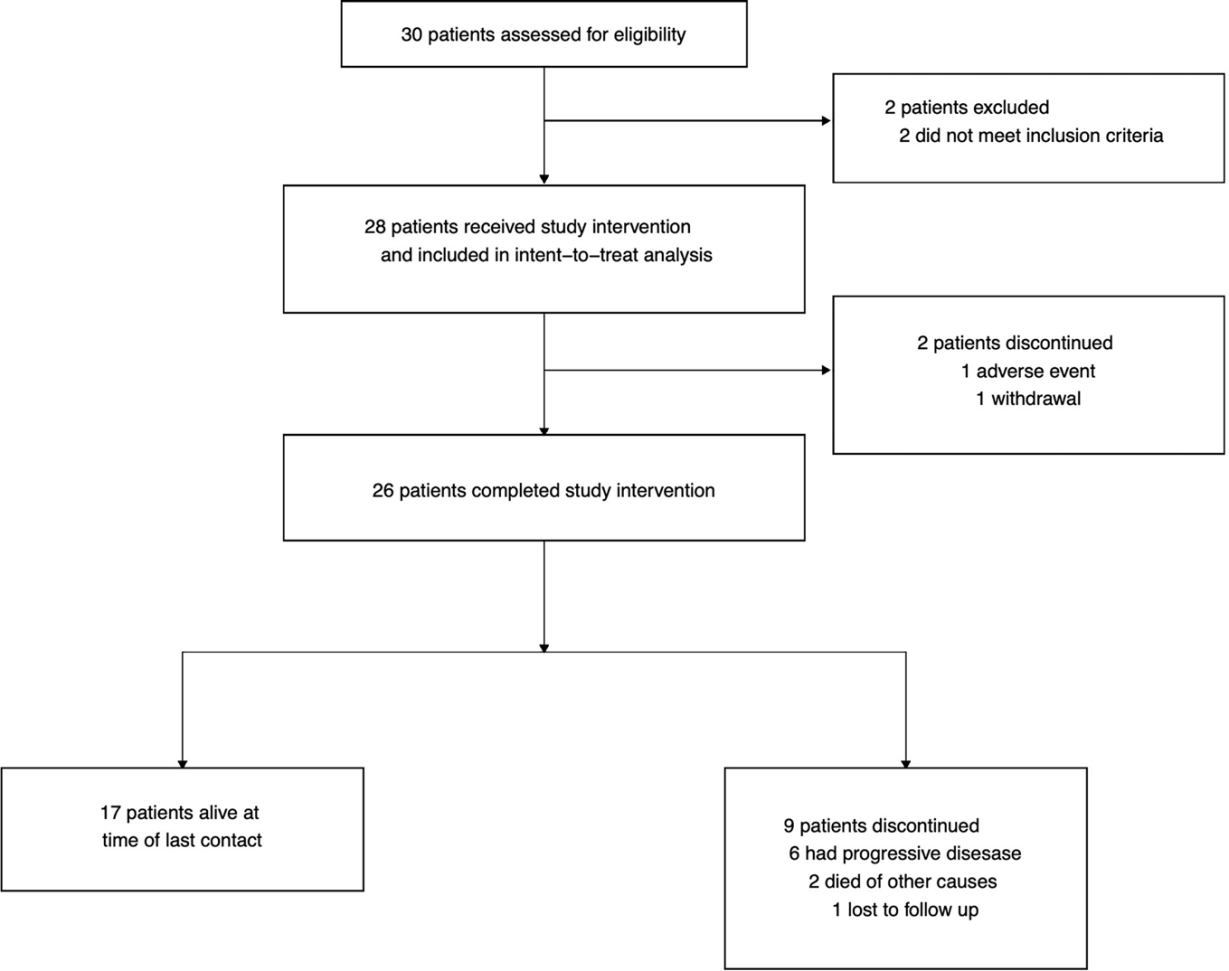
CONSORT diagram.

Thirty patients were assessed for eligibility; 2 were excluded as they did not meet inclusion criteria: one patient due to presence of tumor cells in CSF by cytology at diagnosis, and the other due to a delay in starting radiation therapy (**Figure 1**). A total of 28 patients were enrolled on study between April 2001 and December 2010 across three institutions (Children’s Hospital of Philadelphia, Children’s Hospital of Atlanta, and Lucile Packard Children’s Hospital at Stanford University). Median age at enrollment was 9 ± 0.8 years (range: 2.8 to 17.7 years). Collected patient characteristics are listed in **Table 1**. The study was terminated early due to slow accrual in light of evolving knowledge of molecular subgrouping and risk stratification in medulloblastoma.

**Table 1:** Patient characteristics.

Age at enrollment, median ± SEM (range)	9 ± 0.8 years (33 months to 212 months)
Female, n (%)	9 (32)
Ethnicity	
African American, n (%)	4 (14)
White Hispanic, n (%)	5 (17)
White non-Hispanic, n (%)	19 (68)
Extent of resection	
Gross total resection, n (%)	28 (100)
Subtotal resection, n (%)	0 (0)
Time to radiation therapy, median ± SEM	25 ± 0.9 days
Type of radiation therapy	
Photon radiation, n (%)	28 (100)
Electron radiation, n (%)	0 (0)

SEM, standard error of the mean.

### Safety and Tolerability

Two patients (7%) withdrew from study; one due to toxicity from chemotherapy and the other due to withdrawal of consent. Both patients were included in the intent-to-treat analysis. Eight patients (29%) received all 6 cycles of cisplatin-containing chemotherapy whereas the remainder (20, 71%) had dose reductions, omissions or were changed to carboplatin for renal toxicity, ototoxicity, or parental preference, with approval of the study chair. No dose reductions in regimen B (IV cyclophosphamide, IV and oral etoposide) were required. Of the 25 patients with endocrinologic monitoring data, 15 (60%) required thyroid hormone replacement and 7 (28%) required growth hormone replacement at some point during or after their treatment course. Collected toxicity data are summarized in **Table 2**. There were no grade 5 toxicities.

**Table 2:** Evaluated toxicities.

Toxicity	Number affected (%)
Hearing (n = 28 patients)	
Grade 2	8 (29)
Grade 3	6 (21)
Grade 4	4 (14)
Renal (n = 28 patients)	
Grade 1	1 (4)
Grade 2	7 (25)
Grade 3 or higher	0
Endocrine (n = 25 patients)	
Thyroid hormone replacement	15 (60)
Growth hormone replacement	7 (28)

### Survival

**Figure 2:**
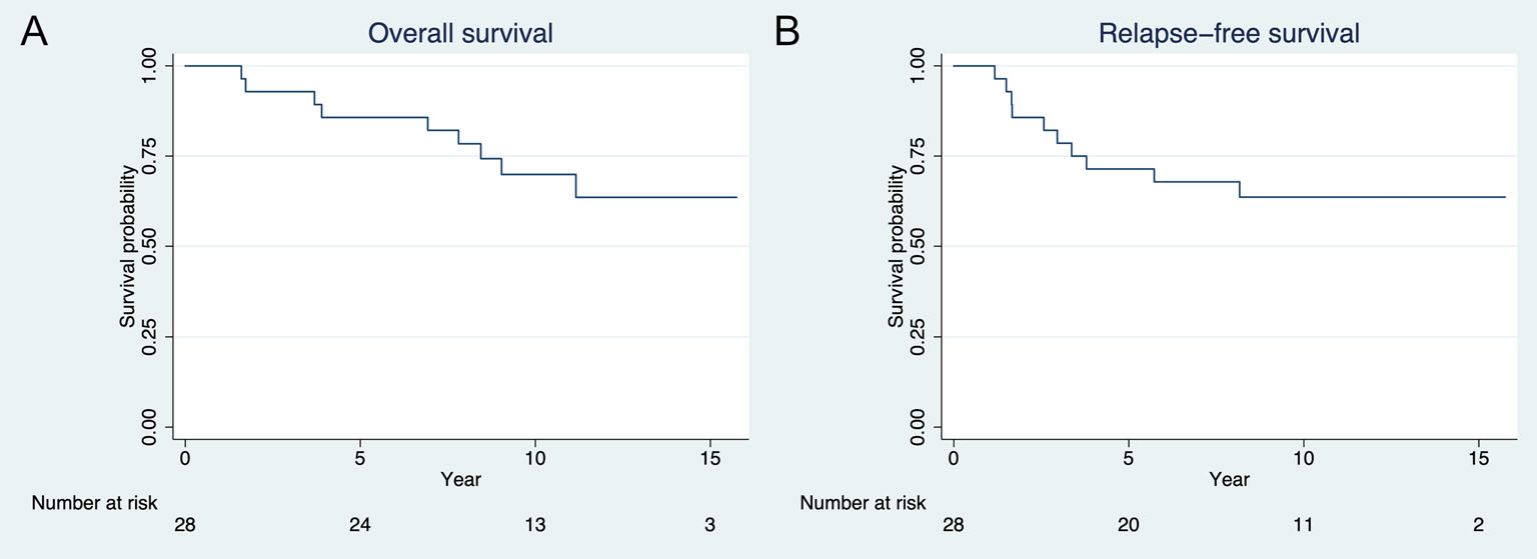
Kaplan-Meier plot of **(A)** overall survival and **(B)** recurrence free survival. Three- and 5-year overall survival are 93% and 86%, respectively. Three- and 5-year relapse-free survival are 79% and 71%, respectively.

The median follow-up time was 140 months (11.7 years; interquartile range 111 to 158 months) by reverse Kaplan-Meier estimator (15). The 3-year RFS and OS were 79% (95% CI 58% to 90%) and 93% (95% CI 74% to 98%), respectively. The 5-year RFS and OS were 71% (95% CI 50% to 85%) and 86% (95% CI 66% to 94%), respectively (**Figures 2A, B**). Nineteen patients (68%) were alive at the time of last contact with no evidence of disease. Seven patients (25%) died of disease, whereas one patient died of secondary glioblastoma and one (who was later diagnosed with a germline *TP53* mutation) of secondary skull base sarcoma.

Eighteen patients (64%) were greater than 7 years of age at enrollment. For this subgroup, the 3- and 5-year OS were 83% (95% CI 68% to 100%) and 78% (95% CI 61% to 100%). Of these 18 patients, 11 (61%) were alive at the time of last contact with no evidence of disease at a median follow up time of 11.7 ± 1.0 years. There was no difference in 3- and 5-year OS when compared to patients less than or equal to 7 years of age or in all patients when stratified by sex.

### Patterns of Relapse

Seven patients (25%) developed relapsed disease: two patients with diffuse or leptomeningeal relapse including the primary site; three with isolated intraspinal tumor; one with exoprimary brain relapse solely in the pituitary; and one patient who initially developed primary site relapse and subsequently disseminated disease.

### Neurocognitive Outcomes

Partial neurocognitive data was available on 15 patients at multiple different time points, limiting complete analysis. Collected data demonstrated mixed changes over time (**Supplementary Figure 1**). One year from the initiation of therapy, one patient showed an increase of greater than 10 IQ points, one patient demonstrated a decrease of greater than 10 IQ points. The remaining six patients from whom data was collected remained within 10 IQ points of their baseline. At 3 years, two patients experienced a decline in greater than 10 IQ points, one remained stable and another had an increase of greater than 10 IQ points from baseline.

## Discussion

In this study, CSI dose reduction to 18 Gy in select patients with average-risk medulloblastoma was associated with similar 3- and 5-year relapse-free and overall survival as well as toxicity profiles compared with historical clinical trials. Considering the burgeoning literature on the long-term effects of chemoradiotherapy in the treatment of pediatric cancers, many studies have sought to risk stratify patients while maintaining efficacy. In this study, there was no significant difference in 3-year RFS in comparison to CCG 9892 (3-year EFS 86% ± SEM 4%); however, there were 4 out of 28 isolated exoprimary relapses (compared to 3 out of 14 in CCG 9892). The Children’s Oncology Group ACNS0331 study has also evaluated CSI dose reduction in patients 3 to 7 years of age with newly diagnosed average-risk medulloblastoma. The data demonstrated inferiority of low dose compared to standard dose craniospinal irradiation in both EFS and OS. However, when post-hoc molecular subtyping was performed, the inferior outcomes in the low-dose CSI arm were largely driven by group 4 tumors (11). In review of the published literature, this is the only clinical study that addresses the utilization of 18 Gy CSI in a patient greater than 7 years of age. These data suggest that reduced dose CSI in select patients with average-risk medulloblastomas should be studied.

Although chemotherapy was relatively well tolerated, 71% of patients on this study had dose reductions of cisplatin or substitutions for carboplatin due to ototoxicity and/or nephrotoxicity, both well-recognized adverse events associated with cisplatin (16–18). Indeed, newer medulloblastoma protocols seeking to limit cisplatin-associated toxicity have reduced the total cumulative dosage in patients with average-risk medulloblastoma without decreases in overall survival (19, 20). In this study, 2 out of 28 patients (7%) developed secondary malignancies. Although one was later diagnosed with a germline *TP53* mutation, this rate is higher than the 3.4% seen for all patients in ACNS0331.

This study is not without limitations. As a single-arm trial with a small sample size, the results are difficult to generalize, and subgroup analyses were not performed. This study took place prior to the widespread implementation of molecular subtyping, which is now essential in medulloblastoma clinical trial risk stratification (21), and ultimately led to early closure prior to meeting accrual goals. Residual tissue for post-hoc testing was limited due to use in correlative studies; in addition, retrospective molecular analysis was not provided for in the protocol, nor is the number of subjects large enough for meaningful comparison, significantly limiting conclusions in this era of integrated molecular diagnostics. Of note, wingless (WNT) pathway activation has been associated with an excellent prognosis in patients with medulloblastoma (22); the concept of dose reduction in this subgroup is currently being studied in clinical trials. While the sample size is small, it is statistically improbable based on age and sex distributions that all patients recruited in this study had WNT-activated tumors (22–24). Thus, the similarity in OS and EFS of this study to others is likely not spurious. The exclusion of anaplastic histologies from average risk medulloblastoma trials was implemented in 2008. Finally, the interpretation of neurocognitive data is significantly impacted by incomplete collection and indeed precludes the analysis of the study’s primary objective. The limited psychometrics implemented in this study were typical of the era and have since been supplanted by more precise and standardized batteries of tests, e.g., the Children’s Oncology Group study ALTE07C1 (25), which was added to ACNS0331 as an amendment in February 2011.

Overall, though the results of the study were somewhat disappointing compared to prior studies, many patients achieved long-term survival without relapse with 18 Gy CSI, potentially limiting late neurocognitive deficits and neurovascular disease and improving quality of life. With the emerging knowledge of differing clinical behaviors by subgroup of medulloblastoma, it is possible that the small differences in outcomes in this study are explained by molecular subtype. Larger, randomized controlled trials with molecular classification and integrated risk adapted staging are needed.

## Publisher’s Note

All claims expressed in this article are solely those of the authors and do not necessarily represent those of their affiliated organizations, or those of the publisher, the editors and the reviewers. Any product that may be evaluated in this article, or claim that may be made by its manufacturer, is not guaranteed or endorsed by the publisher.

## Data Availability Statement

The original contributions presented in the study are included in the article/**Supplementary Material**. Further inquiries can be directed to the corresponding author.

## Ethics Statement

The studies involving human participants were reviewed and approved by participating sites’ respective institutional review boards. Written informed consent to participate in this study was provided by the participants’ legal guardian/next of kin.

## Author Contributions

Study conception and design: JM, JB, IG, BL, PP, PF, MF, and AJ. Acquisition, analysis, and interpretation of data: JM, AM, SP, YL, PF, MF, and AJ. Drafting of manuscript: AM and SP. Critical revision: all authors. All authors contributed to the article and approved the submitted version.

## Conflict of Interest

The authors declare that the research was conducted in the absence of any commercial or financial relationships that could be construed as a potential conflict of interest.

## Publisher’s Note

All claims expressed in this article are solely those of the authors and do not necessarily represent those of their affiliated organizations, or those of the publisher, the editors and the reviewers. Any product that may be evaluated in this article, or claim that may be made by its manufacturer, is not guaranteed or endorsed by the publisher.

## Supplementary Material

Supplementary Figure 1 Collected raw full scale intelligence quotients (FSIQ) for patients on study over time.
