# Supplementary figures and images for: A Pilot Study of Low-Dose Craniospinal Irradiation in Patients With Newly Diagnosed Average-Risk Medulloblastoma
Source: Front Oncol. 2021 Sep 2;11:744739. doi: 10.3389/fonc.2021.744739 (PMC8443797; doi:10.3389/fonc.2021.744739)

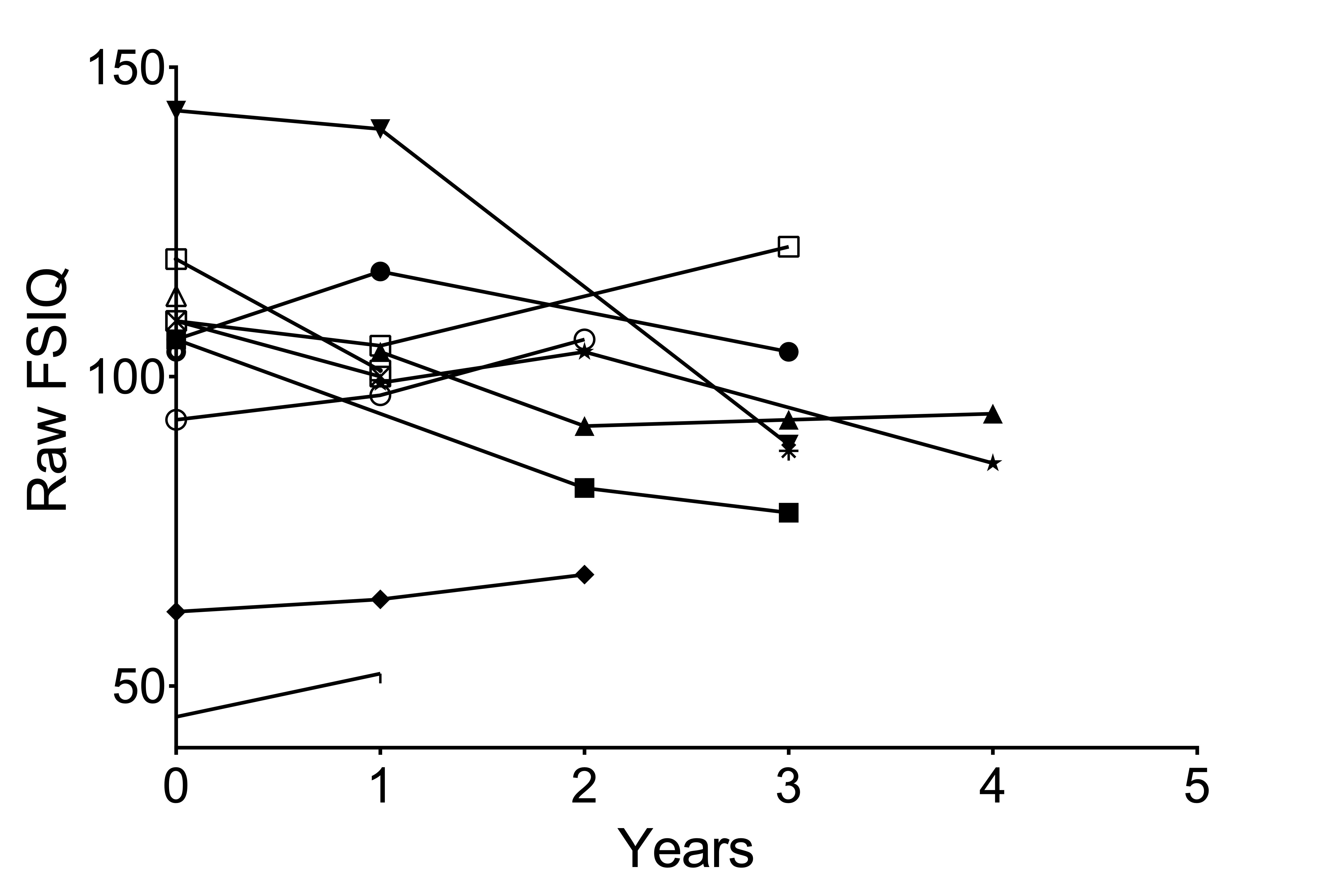

Supplement: Supplementary Figure 1 — Collected raw full scale intelligence quotients (FSIQ) for patients on study over time. [file Image_1.jpg]
